# Protein Aggregation as a Bacterial Strategy to Survive Antibiotic Treatment

**DOI:** 10.3389/fmolb.2021.669664

**Published:** 2021-04-16

**Authors:** Celien Bollen, Liselot Dewachter, Jan Michiels

**Affiliations:** ^1^Centre of Microbial and Plant Genetics, KU Leuven, Leuven, Belgium; ^2^Center for Microbiology, VIB-KU Leuven, Leuven, Belgium

**Keywords:** stress response, amyloid, amorphous aggregate, antibiotic tolerance, persistence, VBNC, dormancy

## Abstract

While protein aggregation is predominantly associated with loss of function and toxicity, it is also known to increase survival of bacteria under stressful conditions. Indeed, protein aggregation not only helps bacteria to cope with proteotoxic stresses like heat shocks or oxidative stress, but a growing number of studies suggest that it also improves survival during antibiotic treatment by inducing dormancy. A well-known example of dormant cells are persisters, which are transiently refractory to the action of antibiotics. These persister cells can switch back to the susceptible state and resume growth in the absence of antibiotics, and are therefore considered an important cause of recurrence of infections. Mounting evidence now suggests that this antibiotic-tolerant persister state is tightly linked to—or perhaps even driven by—protein aggregation. Moreover, another dormant bacterial phenotype, the viable but non-culturable (VBNC) state, was also shown to be associated with aggregation. These results indicate that persisters and VBNC cells may constitute different stages of the same dormancy program induced by progressive protein aggregation. In this mini review, we discuss the relation between aggregation and bacterial dormancy, focusing on both persisters and VBNC cells. Understanding the link between protein aggregation and dormancy will not only provide insight into the fundamentals of bacterial survival, but could prove highly valuable in our future battle to fight them.

## Introduction

Failure of antibiotic treatment has become a worldwide problem due to the prevalence and spread of different bacterial survival mechanisms. One way in which bacteria can survive antibiotic treatment is by becoming resistant through genetic changes that allow bacteria to grow in the presence of the antibiotic, for example, by promoting efflux of the drug, changing the antibiotic target, or directly inactivating the antibiotic (Reygaert, 2018). Apart from surviving antibiotics by acquiring genetic resistance, cells can also protect themselves without acquiring heritable genetic changes. An example of such a non-genetic antibiotic survival mechanism is becoming dormant. Dormant cells are characterized by lower metabolism and a lack of growth (Lennon and Jones, 2011). As antibiotics need active targets (Eng et al., 1991), the shutdown of some important pathways is thought to prevent the antibiotic’s corrupting effects, thereby inducing tolerance (Hu and Coates, 2012; Balaban et al., 2019). A well-known example of dormant cells are persisters. Persisters constitute a small, genetically identical subpopulation of bacteria that are transiently tolerant to antibiotics. They cannot grow in the presence of the antibiotic but can withstand antibiotic pressure as long as they reside in the persister state. These persister cells are most often thought to survive antibiotic treatment by becoming dormant, for example, by lowering ATP levels and by inhibiting important macromolecular processes like transcription and translation (Dewachter et al., 2019; Wilmaerts et al., 2019b). However, persistence has also sporadically been associated with active mechanisms like the activity of antibiotic efflux pumps and DNA repair (Nguyen et al., 2011; Orman and Brynildsen, 2013a; Völzing and Brynildsen, 2015; Pu et al., 2016). Despite being dormant, persisters can easily resume growth when antibiotics are removed (Balaban et al., 2019; Wilmaerts et al., 2019a). This regrowth has been implicated in the chronic nature of infections (Dhar and McKinney, 2010; Mulcahy et al., 2010; Goneau et al., 2014; Schumacher et al., 2015).

Besides persistence, other dormant bacterial phenotypes like the viable but non-culturable (VBNC) state exist (Xu et al., 1982). VBNC cells remain metabolically active, but they have lost the ability to grow on standard medium that would otherwise support their proliferation (Oliver, 1993). This dormancy protects VBNC cells from antibiotic and other stresses (Nowakowska and Oliver, 2013). Contrary to persisters, VBNC cells do not resume growth when provided with fresh medium, but instead, they need a specific factor to resuscitate (Li et al., 2014). Although these resuscitation factors are not always known (Yamamoto, 2000), it appears as though at least some VBNC cells can resuscitate *in vivo* (Colwell et al., 1996) and cause recurrent infections (Pai et al., 2000; Rivers and Steck, 2001).

Despite the difference in resuscitation, persisters and VBNC cells also share some properties. They are both tolerant to antibiotics (Nowakowska and Oliver, 2013; Balaban et al., 2019) and reside in a dormant state with no or slow growth (Xu et al., 1982; Balaban et al., 2004), a low metabolism (Shleeva et al., 2004; Amato et al., 2014), and reduced energy production (Dörr et al., 2010; Verstraeten et al., 2015; Zhao et al., 2016). Moreover, persisters and VBNC cells also show similarities regarding their formation, suggesting a link between them. Persisters and VBNC cells can both be generated stochastically in unstressed exponential phase cultures (Balaban et al., 2004; Orman and Brynildsen, 2013b). However, more often, they are induced by environmental stresses. Some examples of stresses that induce both dormant phenotypes are nutrient (Betts et al., 2002; Mishra et al., 2012), oxidative (Wu et al., 2012; Li et al., 2014), osmotic (Roth et al., 1988; Murakami et al., 2005), acid (Cunningham et al., 2009; Hong W. et al., 2012), and temperature stress (Oliver et al., 1991; Cardoso et al., 2010). Additionally, both persistence and the VBNC state are linked to the general stress response (Boaretti et al., 2003; Murakami et al., 2005), toxin-antitoxin modules (Moyed and Bertrand, 1983; Korch and Hill, 2006), and protein aggregation (Leszczynska et al., 2013; Mordukhova and Pan, 2014; Pu et al., 2019; Yu et al., 2019; Cesar et al., 2020; Dewachter et al., 2021; Huemer et al., 2021). Persisters and VBNC cells thus share many similarities. Therefore, it is hypothesized that they represent different stages of the same dormancy program with different dormancy depths; persisters and VBNC cells reside in a shallow and deep dormant state, respectively (Li et al., 2014; Ayrapetyan et al., 2015; Kim et al., 2018; Pu et al., 2019; Dewachter et al., 2021).

**FIGURE 1:**
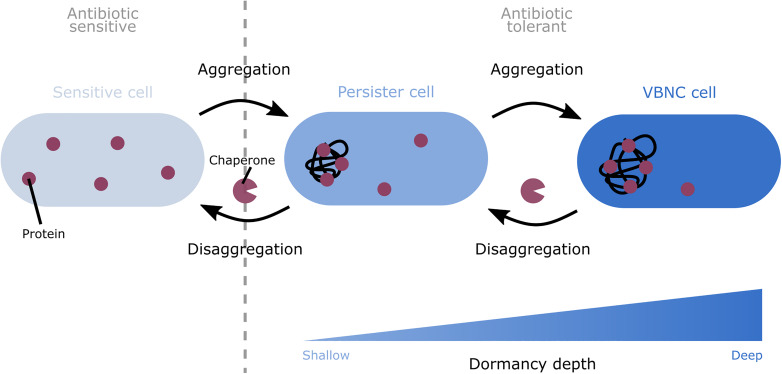
A model depicting the role of protein aggregation in the formation and awakening of dormant cells. Progressive protein aggregation is proposed to induce the shift from sensitive to dormant cells. Aggregation can induce the switch from sensitive cells to the shallowly dormant persister state. Further development of the aggregates can cause a shift from persister cells to the deeper dormant VBNC state. This aggregation-induced dormancy renders cells tolerant to antibiotics. This tolerance is likely caused by the sequestration of proteins in the cell, thereby shutting down different important cellular pathways. To wake up again, these dormant cells likely first need to remove the aggregates. To perform this disaggregation, bacteria make use of chaperones.

Recently, experimental support for this hypothesis has emerged suggesting that both persistence and the VBNC state are linked to protein aggregation and that progressive aggregation can drive the development from persistence to the VBNC state (Figure 1) (Pu et al., 2019; Dewachter et al., 2021). Indeed, previous work also demonstrated a link between aggregation and persistence (Leszczynska et al., 2013; Mordukhova and Pan, 2014; Pu et al., 2019; Yu et al., 2019; Dewachter et al., 2021; Huemer et al., 2021). In this review, we elaborate on the steadily growing number of studies linking protein aggregation and persistence. Additionally, we discuss how aggregation could induce dormancy in general.

## Protein Aggregation in Bacteria

### Formation, Features, and Consequences of Protein Aggregates

For a cell, the amount of proteins that adopts the native state is critical as only correctly folded proteins function properly. This amount depends on the balance between the speed of translation, the rate of protein folding, and the stability of that fold (Sabate et al., 2010). When this balance is disturbed, proteins can unfold or misfold, causing their aggregation-prone regions to be exposed. These aggregation-prone regions are hydrophobic stretches that trigger protein aggregation when they are exposed (Rousseau et al., 2006). They do this by interacting with aggregation-prone regions of other non-native proteins and forming intermolecular β-sheets in a dose-dependent manner (Bednarska et al., 2013).

**FIGURE 2:**
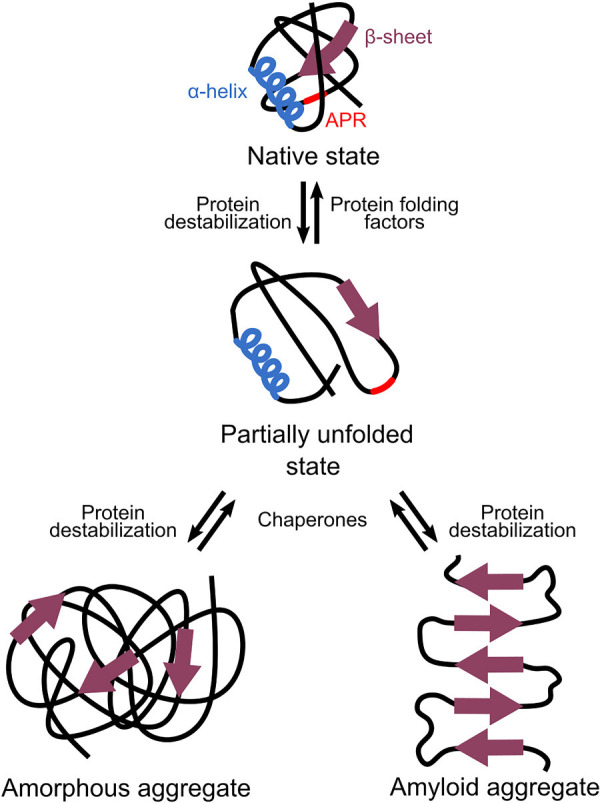
Different types of protein aggregations. When proteins are—at least partially—unfolded or misfolded, they can expose their aggregation-prone regions (APRs). Interaction of APRs of different proteins results in the formation of intermolecular β-sheets that cause aggregation. Amyloid aggregates are highly ordered as their β-sheets run perpendicular to the central axis of the aggregate. Amorphous aggregates also contain some β-structures but lack this long-range order.

Two different classes of protein aggregates exist: amyloid and amorphous aggregates (Figure 2). In amyloid aggregates, the intermolecular β-sheets run perpendicular to the central axis of the aggregate, which gives them their highly ordered structure (Sunde and Blake, 1997). Next to amyloids, amorphous aggregates or inclusion bodies exist. These amorphous aggregates also contain some amyloid-like β-structures, but they miss the long-range order. This makes them unstructured in electron microscopic images (Wang et al., 2008).

The presence of amorphous or amyloid aggregates is often linked to detrimental effects, such as loss of function of the aggregated proteins (Chiti and Dobson, 2006). In extreme conditions of proteome-wide aggregation induced by frequently occurring aggregation-prone regions, this extensive loss of function can even become lethal (Bednarska et al., 2015; Khodaparast et al., 2018). Next to provoking loss of function, amyloid aggregates are also directly associated with cytotoxicity. This toxicity is most often caused by soluble oligomers that precede the formation of amyloids but not by the more inert mature amyloids themselves (Bucciantini et al., 2002). A possible mechanism by which these oligomers induce toxicity and cell death involves membrane damage and permeabilization (Bednarska et al., 2013). In contrast to amyloids, amorphous aggregates are generally not toxic (Bednarska et al., 2013).

Despite all these negative effects, the presence of aggregates is not always detrimental as some proteins remain active in amorphous or amyloid aggregates (Arié et al., 2006). Additionally, certain proteins reach their specific function only when they are structured in amyloids (Chiti and Dobson, 2006). For example, functional amyloids are needed for the robustness and adherence of biofilms, the functionality of specific toxins, and the formation of spores (Garland and Buckley, 1988; Austin et al., 1998; Bednarska et al., 2013). Due to their lower level of organization, amorphous aggregates are not related to these new functionalities (Bednarska et al., 2013). Another beneficial effect of aggregates is their ability to protect the cell against stress (Leszczynska et al., 2013; Mordukhova and Pan, 2014; Govers et al., 2018; Pu et al., 2019; Yu et al., 2019; Dewachter et al., 2021; Huemer et al., 2021). It is not known yet if this increased stress tolerance is a general property or if it is linked to a specific type and/or composition of aggregates.

### Induction, Prevention, and Removal of Protein Aggregates

Since proteins need to be at least partially unfolded or misfolded to aggregate (Uversky and Fink, 2004), aggregation is promoted by increasing the amount of non-native proteins. This can be done by increasing the amount of newly-formed, unfolded polypeptides by increasing translation or decreasing the rate of protein folding (Tartaglia et al., 2009). Another way to trigger aggregation is by destabilizing the native fold (Chiti et al., 2000). Many destabilizing factors exist such as changes in the protein sequence caused by genetic mutations (Hurle et al., 1994), modifications due to oxidative stress (Dahl et al., 2015), or mistranslation (Drummond and Wilke, 2008). Additionally, protein unfolding or misfolding can also be triggered by external stresses such as heat (Litvinovich et al., 1998), high pressure (Ferrão-Gonzales et al., 2000), extreme pH (Guijarro et al., 1998), moderate concentrations of organic solvents or alcohols (Chiti et al., 1999), and osmotic (Schramm et al., 2020) and oxidative stress (Mirzaei and Regnier, 2008).

Because aggregation can render proteins dysfunctional, cells try to minimize the amount of non-native proteins through several complementary approaches. First, cells limit the amount of aggregation-prone proteins by controlling transcription, translation, and degradation even more strictly than for non-aggregation-prone proteins (Gsponer and Babu, 2012). Second, cells already start to fold their proteins during translation which minimizes the amount of unfolded peptides in the cytoplasm. Co-translational folding has been shown to be dependent on RNA structure and the presence of rare codons, which induce pauses during translation. These pauses then allow the cell to fold proteins correctly (Purvis et al., 1987; Sabate et al., 2010). Furthermore, specialized chaperones aid the folding of proteins by binding and release cycles that are repeated until the native state is reached (Hartl et al., 2011; Bhuwan et al., 2017). The three major bacterial chaperone complexes are trigger factor, the DnaK-DnaJ-GrpE, and the GroEL-GroES complexes (Sabate et al., 2010). These chaperones can work both independently and cooperatively to fold proteins correctly (Hartl, 1996; Deuerling et al., 1999). The importance of these chaperones is reflected in their conservation among bacteria, archaea, and eukaryotes (Powers and Balch, 2013).

Despite the cell’s efforts to make correctly folded proteins, some proteins will still fold wrongly and aggregate. To remove these aggregates, different chaperones often work together. After disaggregation, proteins can be refolded and reused. However, when the disaggregated proteins are damaged or unneeded, they will be degraded by proteases (Schramm et al., 2020). Taken together, cells will inevitably encounter the formation of non-native proteins and aggregates at some point. The amount of aggregation that the cell experiences depends on a variety of factors that influence the very delicate balance between proteins in the soluble and aggregated state (Carrió and Villaverde, 2001).

## The Role of Protein Aggregation in Bacterial Dormancy

### Protein Aggregation and Dormancy Correlate at the Single-Cell and Population Level

Despite the detrimental effects that are commonly associated with aggregation, the presence of aggregates could also be beneficial since it has repeatedly been suggested to protect bacteria against antibiotic stress. An increasing number of studies have linked protein aggregation to different forms of bacterial dormancy, in particular persistence. Because of the tight association between both processes, we and others have hypothesized that protein aggregation drives dormancy development. This hypothesis is supported by the observation that in *Escherichia coli* persisters and VBNC cells more often contain aggregates than non-dormant cells (Pu et al., 2019; Yu et al., 2019; Cesar et al., 2020) and that protein aggregation in these dormant cells occurs more intensely (Dewachter et al., 2021). Moreover, the intensity of aggregation, measured by expression of IbpA-msfGFP and therefore the amount of proteins that are aggregated, appears to be correlated to dormancy depth at the single-cell level; shallowly dormant persisters carry low intensity aggregates, while deeper dormant VBNC cells contain more intense aggregates (Dewachter et al., 2021). However, not all cells with protein aggregates are dormant (Dewachter et al., 2021), which suggests that a certain level or threshold of aggregation is needed in the cells to shift to the dormant state. As aggregates were shown to develop gradually (Yu et al., 2019; Dewachter et al., 2021), the correlation between aggregate intensity and dormancy depth implies that a general dormancy program may exist in which progressive protein aggregation could induce the shift from the susceptible to the persister state and from the persister to the VBNC state (Figure 1) (Dewachter et al., 2021).

Besides the tight association between protein aggregation and bacterial dormancy demonstrated at the single-cell level, further support for the association and potentially causal relation between aggregation and dormancy was found at the population level. In clinically isolated *Staphylococcus aureus* cultures, persisters were shown to accumulate insoluble proteins (Huemer et al., 2021). Moreover, multiple studies performed with *E. coli* observed that influencing aggregation causes a similar change in dormancy, thereby revealing a direct link between them. For example, decreasing aggregation by buffering the pH of the growth medium or by adding low levels of osmolytes also decreased the persister level (Leszczynska et al., 2013). Additionally, suppressing aggregation by administering chloramphenicol reduced both aggregation and dormancy (Pu et al., 2019). On the other hand, when aggregation was increased by adding acetate, the persister level also rose (Leszczynska et al., 2013; Mordukhova and Pan, 2014). Other conditions that induce aggregation like high temperatures or the addition of streptomycin or hydrogen peroxide augmented dormancy as well (Pu et al., 2019). Besides these external triggers, genetic factors were also shown to influence both aggregation and dormancy. For example, overexpression of the persister gene *obgE*, which encodes a small GTPase that plays a role in ribosome assembly and functioning (Feng et al., 2014), not only accelerated persister development, but also triggered aggregation and the formation of VBNC cells (Verstraeten et al., 2015; Dewachter et al., 2021). Besides *obgE*, overexpression of *metA*, which encodes an unstable protein involved in the biosynthesis of methionine (Rowbury, 1965), resulted in more aggregation of this protein at high temperatures. This increased aggregation was accompanied by an increase in persistence. Stabilizing the MetA protein not only reduced its aggregation, but also lowered the persister level (Mordukhova and Pan, 2014). Consequently, different studies have found a direct association between aggregation and the induction of persistence and/or the VBNC state at both the single-cell and the population level.

Possibly, aggregation is more prevalent in dormancy development than currently thought because different studies have separately shown that aggregation and dormancy are induced by the same factors. Entry into stationary phase not only induces progressive aggregation (Kwiatkowska et al., 2008), but also different depths of dormancy (Pu et al., 2019; Yu et al., 2019; Cesar et al., 2020; Dewachter et al., 2021). This increased aggregation and dormancy in stationary phase may be caused by nutrient deprivation and consequently ATP depletion (Pu et al., 2019). Indeed, the ATP level in a population enriched in persister cells was shown to be reduced by 50% (Huemer et al., 2021). Moreover, ATP depletion is linked to the formation of dormant cells (Dörr et al., 2010; Kwan et al., 2013; Verstraeten et al., 2015; Zhao et al., 2016; Wilmaerts et al., 2018; Huemer et al., 2021) and protein aggregation (Pu et al., 2019; Dewachter et al., 2021). Additionally, acid stress is also known to induce aggregation (Kern et al., 2007), persistence (Hong S.H. et al., 2012), and the VBNC state (Cunningham et al., 2009). Another stress that is linked to the induction of aggregation (Schramm et al., 2020) and dormancy (Roth et al., 1988; Murakami et al., 2005) is osmotic stress. Reducing osmotic stress by adding low concentrations of osmolytes can resuscitate VBNC cells (Roth et al., 1988) and inhibit aggregation (Diamant et al., 2001). Furthermore, oxidative stress (Arana et al., 1992; Mirzaei and Regnier, 2008; Hong S.H. et al., 2012) and heat stress (Oliver, 2000; Murakami et al., 2005; Schramm et al., 2019) also induce aggregation, persistence, and the VBNC state. Finally, induction of proteotoxic mistranslation by exposing bacteria to sub-MIC concentrations of aminoglycosides like gentamycin and streptomycin (Davies et al., 1964) or by exposing them to trimethoprim, which interrupts the folate metabolism (Huang et al., 1997), increases persistence (Kwan et al., 2013) and aggregation (Laskowska et al., 2002; Lindner et al., 2008; Goltermann et al., 2013). Because a wide variety of factors influence both aggregation and dormancy, protein aggregation could possibly be a widespread phenomenon that is related to the onset of dormancy over many different inducing conditions.

### Protein Aggregation Is Hypothesized to Induce Dormancy by Shutting Down Important Cellular Pathways

The clear correlation between aggregation and dormancy suggests that aggregation could be responsible for the formation of dormant cells. Indeed, it has been hypothesized that aggregation induces dormancy by shutting down different important cellular pathways (Figure 1).

Protein aggregates present in dormant cells contain a wide variety of proteins of important pathways like energy production and translation (Leszczynska et al., 2013; Pu et al., 2019; Yu et al., 2019; Dewachter et al., 2021; Huemer et al., 2021). Although antibiotic targets are also present in the aggregate, their direct sequestration is probably not important for the induction of tolerance in *E. coli* (Dewachter et al., 2021). Instead, the aggregation and consequent loss of function of multiple proteins may lead to a gradual shutdown of cellular metabolism, which then causes dormancy and tolerance. The hypothesis that inhibition of important pathways may induce dormancy is supported by the observation that lowering transcription or translation by toxins or the addition of antibiotics also induces persistence (Kwan et al., 2013; Cheverton et al., 2016). Although inhibition of transcription or translation by antibiotics, toxins, and aggregation might work differently, it shows that the shutdown of important pathways can indeed be an important cellular mechanism to induce antibiotic tolerance. Moreover, as it is hypothesized that aggregation needs to reach a certain threshold before a specific dormancy depth can be induced, this inhibition of important pathways might be the trigger to switch to a deeper dormant state.

### Disaggregation Appears to Be a Prerequisite for Growth Resumption

When aggregation-induced dormant cells resume growth, the aggregate is being removed suggesting that disaggregation is needed for awakening (Figure 1) (Pu et al., 2019; Yu et al., 2019; Cesar et al., 2020; Huemer et al., 2021). Different chaperones play an important role in this disaggregation process. The chaperones DnaK and ClpB were shown to colocalize with the aggregates of *E. coli* persister cells prior to their awakening, but failed to do so in VBNC cells that remained dormant (Pu et al., 2019). Additionally, impairing the disaggregation activity of DnaK, and to a minor extent also the activity of ClpB, increased dormancy in general but reduced regrowth suggesting problems with awakening (Pu et al., 2019; Cesar et al., 2020). This indicates that disaggregation by chaperones such as DnaK, and possibly also ClpB, could be important for aggregation-induced dormant cells to resume growth. Moreover, as it was shown that the FtsZ protein can be refolded and resume its function after disaggregation (Yu et al., 2019), it is hypothesized that disaggregation is required to recover the proteins inside the aggregate to restart important cellular pathways. However, as this reactivation was only investigated for a single protein, further confirmation is still needed to see if the reactivation of aggregated proteins or the removal of the aggregates itself is important for awakening. However, the causality between disaggregation and awakening has not been fully established yet. It therefore remains possible that cells wake up by replenishing their energy levels and that the observed disaggregation is merely a side effect of the increased levels of ATP, which is needed for chaperone activity.

## Conclusion and Future Perspectives

Even though protein aggregates are mostly known for their detrimental effects, they may also protect cells against antibiotics by inducing dormancy. Indeed, both persistence and the VBNC state, which are tolerant phenotypes with different dormancy depths, have been linked to protein aggregation (Leszczynska et al., 2013; Mordukhova and Pan, 2014; Pu et al., 2019; Yu et al., 2019; Cesar et al., 2020; Dewachter et al., 2021; Huemer et al., 2021). Additionally, as aggregates were shown to develop gradually and as the intensity of aggregation has been correlated to different dormancy depths, it has been suggested that progressive protein aggregation could induce different depths of dormancy (Dewachter et al., 2021). First, aggregation may cause sensitive cells to switch to the shallowly dormant persister state. Further development of the aggregates subsequently drives these persister cells into a deeper dormant VBNC state. At the mechanistic level, it is hypothesized that aggregation leads to the sequestration of important cellular proteins, which leads to the shutdown of cellular metabolism and consequently also to dormancy (Leszczynska et al., 2013; Pu et al., 2019; Yu et al., 2019; Dewachter et al., 2021). To resume growth, it is suggested that dormant cells first remove aggregates (Pu et al., 2019). As a protein’s functionality can be recovered following disaggregation (Yu et al., 2019), such disaggregation may lead to a restart of important cellular pathways, thereby potentially explaining why disaggregation is a prerequisite for growth resumption.

Even though the above explanation for the link between protein aggregation and bacterial dormancy seems appealing, there are still some important unanswered questions. First, despite the frequently confirmed correlation between aggregation and dormancy, conclusive proof for a causal relationship between both processes is still missing. Second, in case such a relationship exists, the molecular mechanism by which protein aggregation drives dormancy development needs to be resolved. Additionally, since not all cells that carry protein aggregates are dormant, it is hypothesized that a certain threshold of aggregation is needed to induce dormancy. It therefore needs to be investigated what this specific threshold is, if it is reached stepwise or gradually and if it depends on the composition of the aggregate. Third, the fate of disaggregated proteins needs to be investigated further to see if they are refolded and reused or if they are degraded. Clearly, addressing these current research gaps will require advanced single-cell approaches. For example, developments in microfluidics and physiological reporters will make it possible to track the aggregation and disaggregation process in real time in a high-throughput manner and correlate it to changes in cell physiology. Clearly, many questions are left unanswered. However, a link between aggregation and dormancy has been repeatedly demonstrated and may lead to breakthroughs in both the dormancy and the aggregation fields. Furthermore, if protein aggregation is revealed to form the link between several redundant persister pathways that are already known today, it might be an important starting point for the development of highly-needed anti-persister therapies in the future.

## Author Contributions

CB wrote the original draft. All the authors performed review and editing.

## Conflict of Interest

The authors declare that the research was conducted in the absence of any commercial or financial relationships that could be construed as a potential conflict of interest.
